# GFORCE-PD still going strong in 2016

**DOI:** 10.1038/npjparkd.2016.14

**Published:** 2017-02-23

**Authors:** Malin Parmar, Jun Takahashi, Lorenz Studer, Roger A Barker

**Affiliations:** 1 Wallenberg Neuroscience Center, and Lund Stem Cell Center Lund University Lund, Sweden; 2 Center for iPS Cell Research and Application (CiRA) Kyoto Univerisity Kyoto, Japan; 3 Center for Stem Cell Biology, Memorial Sloan Kettering Cancer Centre, New York, NY, USA; 4 John van Geest Centre for Brain Repair, Department of Clinical Neuroscience, University of Cambridge, Cambridge, UK

## Abstract

In 2014, a new initiative was undertaken by groups working on plans for the transplantation of stem-cell-based derived dopaminergic neurons for treating Parkinson’s disease patients. This GForce-PD group held its annual meeting on 18–19 April 2016 in Chicago at Rush University to discuss their progress and the challenges that the translation of this experimental therapy still faces. Over 2 days, the key issues were discussed around the cell lines that will be used, the differentiation protocols, preclinical testing, GMP-adaptation, and cell manufacturing to allow first in human clinical trials, which are anticipated to start in 2017–2018. GForce-PD members also discussed how they can improve outreach and be of better service to the Parkinson's disease (PD) community and help them to make the best possible decisions when pursuing stem cell treatments.

## Introduction

In 2014, a group of scientists and clinicians met for the first time to focus on accelerating the translation of stem-cell-based therapies to the clinic for first in human trials.^1^ The rationale for this was the belief that collective work around developing the necessary protocols to allow cells to get to patients would be facilitated and enhanced by such a combined effort. In addition, a further strength of the initiative would be to ensure that any such trial is only undertaken when all the necessary elements are in place, both in terms of regulatory approval and preclinical evidence of graft survival, safety and efficacy.^2^ The group that met in London UK became known as GForce-PD and includes leading groups from Europe funded by the EU, TransEuro (http://www.transeuro.org.uk/) and NeuroStemCellRepair (http://www.neurostemcellrepair.org/); the NYSTEM (New York State Stem Cell Science) consortium led by Lorenz Studer and Viviane Tabar (https://www.mskcc.org/research-areas/programs-centers/new-york-state-stem-cell-science-consortia), and the CiRA group of Jun Takahashi (https://www.cira.kyoto-u.ac.jp/e/research/takahashi_summary.html), along with others linked to this work through their role in patient organizations as well as relevant funding agencies. The second of these meetings took place last year in New York at the Memorial Sloan Kettering Cancer Center, and the most recent took place in Chicago, hosted by Jeff Kordower on the 18–19 April.

At this latest meeting it was clear that great progress had been made over the previous 12 months in terms of moving closer to the clinic for a first in human trial with stem-cell-derived dopaminergic cells, as well as helping define the best way to discriminate between therapies that are of merit versus those that are not. This was illustrated by discussing the recently reported planned trial using parthenogenetic embryonic stem cells for a trial in 12 PD patients in Melbourne run by the company ISCO—a commentary of which can be found in ref. 3.

Key points discussed:

1. The development of a stem-cell-based therapy for clinical application is not straightforward, especially at the regulatory level. This was well illustrated by the talk of Jane Lebkowski from Asterias Biotherapeutics who led us through the long route by which their stem-cell-derived neural progenitor product was eventually taken to clinical trials in patients with spinal cord injury.
2. TRANSEURO is an EU funded clinical trial looking at better understanding how to optimize the transplantation of human fetal ventral mesencephalic tissue into patients with PD. Roger Barker summarized the program and the problems that have arisen during its long evolution, as well as commenting that 11 transplants have now been successfully completed in the last 11 months. The design of this trial has helped define the tools needed for any stem cell studies in patients, and this was discussed in terms of assessment tools, patient selection and primary end points.
3. NeuroStemCellRepair is another EU consortium that has as part of its remit to deliver a GMP compatible dopaminergic cell derived from a stem cell source for use in patients with PD. Malin Parmar described how the protocols have now been modified to be of the necessary GMP standard, without them compromising the differentiation efficiency and functional efficacy of the cells so produced.^4,5^ This work is expected to lead to a filing for a clinical trial in 2018.
4. Jun Takahashi also explained how their iPS programme for PD has now been modified as part of the CiRA programme of work on regenerative medicine. Their work showed how the protocol for generating iPS-derived dopamine cells has improved in terms of the efficacy and authenticity of the cells they now are testing in non-human primate xenografts.^6^ This will lead onto trials in the near future using allogeneic (not autologous) iPS cells as the donor source. In particular they plan to use so called HLA-homozygous iPSCs, which provide a ‘hybrid’ between personalized medicine and off-the-shelf therapies. It is anticipated that 75 lines will be needed to cover ~80% of the Japanese population from this immunological perspective.
5. Lorenz Studer summarized the last 12 months of work that his group has undertaken using the WA09/H9 ES cell line in terms of modifying their differentiation protocol.^7,8^ They have now locked down this protocol and started GMP production of the cells. The team, sponsored by NYSTEM have a meeting scheduled with the FDA in May 2016 to discuss the IND enabling studies planned for their trial. They anticipate filing for a clinical trial to begin late in 2017.
6. In addition, a number of other presentations on stem cells were given:
  - Kwang Soo Kim (Harvard University) discussed his work on iPS cell derivation and the processes limiting and controlling reprogramming;^9^
  - Ernest Arenas (Karolinska Institute) discussed his recent findings on midbrain development in the mouse and human using single-cell transcriptomics;^10^
  - Tilo Kunath (Edinburgh University) presented his data on the genetic stability of different ES cell lines,^11^ as well as ways to optimize and monitor the dopaminergic differentiation protocol.^11^
7. Other aspects of neural transplantation were discussed, including Jeff Kordower’s presentation on some recent post mortem findings in a patients grafted with fetal VM tissue as part of the original NIH funded trials. Claire Henchcliffe, with the assistance of Cyndy McRae, and the team at Weill Cornell Medical College, was able to identify and meet with a number of patients who had participated in these trials. She presented very long-term follow-up data on their current clinical status and neuroimaging findings almost 2 decades after their original fetal grafts.
8. Finally, a number of other key issues were discussed:
  - Inmaculada de Melo-Martin (Weill Cornell Medical College) presented a discussion on some of the major ethical issues in trials of the type proposed, including the informed consent process, crafting appropriate inclusion and exclusion criteria for subject selection, and the need for long-term follow-up;^12^
  - Johann Thaheld discussed intellectual property and aspects of commercialization that need to be considered, if any of these therapies are to become mainline treatments for PD;
  - Patrik Brundin (van Andel Institute) discussed the possible role of GForce-PD in commenting on trials (both to the scientific and patient community) that seem to pursue practices that are different to those advocated in the International Society for Stem Cell Research guidelines and adopted and implemented by GForce-PD members. His presentation spurred a discussion on how GForce-PD can enhance interactions with the public and engage with the patient community to connect, inform, and educate on the importance of well-planned and scientifically sound clinical trials using stem cells. Similarly, the group thought that informing clinicians about the status of ongoing work is necessary and relevant to their ability to counsel patients and respond to potential inquiries about stem cell trials.
  - And last, the group decided to nominate a steering committee that will shape the future of this group, with members chosen from each of the relevant parties that currently make up GForce-PD.

Over a short one-and-a-half-day period all these various topics were presented and vigorously discussed in a spirit of transparency, collaboration, and passion. Indeed, this meeting once more encapsulated so much of what drives early-stage translational medicine—namely the capacity to work together for the common good by allowing open debate between those with expertize in the basic science; clinical trials and practice; those knowledgeable in regulatory oversight by national/international agencies; and with attention to patient experiences and expectations. All of which augurs well as we plan for the 4th Annual G FORCE-PD meeting in 2017 to be held in Kyoto, Japan.

Finally, with the progress made by all teams in terms of GMP adaptation and cell manufacturing, it is clear that stem-cell-based therapies for PD are only very few years away from the first clinical trials. Although the route to the clinic can seem like a slow and windy road, it is important to remember that the process by which science becomes medicine should be designed to minimize harm and maximize benefit (ref http://www.closerlookatstemcells.org/stem-cells-and-medicine/nine-things-to-know-about-stem-cell-treatments). This is essential so as to ensure that the scientific, clinical and patient community’s enthusiasm to harness the promise of stem cell therapies, is tempered to avoid premature clinical applications which has the potential to derail this whole field of regenerative medicine and place patients at risk.
